# Supplementary material for: Hyperandrogenic Symptoms Are a Persistent Suffering in Midlife Women with PCOS; a Prospective Cohort Study in Sweden
Source: Biomedicines. 2022 Dec 30;11(1):96. doi: 10.3390/biomedicines11010096 (PMC9855793; doi:10.3390/biomedicines11010096)
Supplement: Supplementary file 1 [file biomedicines-11-00096-s001.zip › biomedicines-2081527-supplementary.pdf]

Table S1. Biochemical hyperandrogenism analysed by weight class. Controls vs women with PCOS.

	**Controls** n (%)	**PCOS** n (%)	*p*-value
**Normal weight**	n=21	n=21	
Testosterone, ng/mL, median (min, max)	0.27 (0.13, 0.51)	0.34 (0.20, 0.59)	0.012
SHBG, mmol/L, median (min, max)	71.0 (38.0, 142.0)	57.5 (35.0, 110.0)	0.148
FAI, median (min, max)	0.004 (0.00, 0.01)	0.006 (0.00, 0.01)	0.010
**Overweight**	n=13	n=36	
Testosterone, ng/mL, median (min, max)	0.32 (0.13, 0.70)	0.36 (0.02, 1.29)	0.402
SHBG, mmol/L, median (min, max)	76.0 (32.0, 125.0)	43.0 (19.0, 94.0)	0.005
FAI, median (min, max)	0.005 (0.00, 0.01)	0.009 (0.00, 0.02)	0.003
**Overweight and obese**	n=18	n=106	
Testosterone, ng/mL, median (min, max)	0.35 (0.13, 0.81)	0.35 (0.02, 1.29)	0.791
SHBG, mmol/L, median (min, max)	59.0 (27.0, 125.0)	39.0 (6.4, 108.0)	0.004
FAI, median (min, max)	0.007 (0.00, 0.01)	0.009 (0.00, 0.06)	0.027

Data were not normally distributed. Mann-Whitney U-test was used.
